# Complete response of metastatic papillary renal cell carcinoma with inferior vena cava tumor thrombus to nivolumab plus cabozantinib

**DOI:** 10.1002/iju5.12638

**Published:** 2023-09-05

**Authors:** Michikata Hayashida, Yuji Miura, Takeshi Yamaguchi, Makoto Tanaka, Taro Yamanaka, Kohji Takemura, Suguru Oka, Kazushige Sakaguchi, Shinji Ito, Shinji Urakami

**Affiliations:** ^1^ Department of Urology Toranomon Hospital Tokyo Japan; ^2^ Department of Medical Oncology Toranomon Hospital Tokyo Japan; ^3^ Department of Pathology Toranomon Hospital Tokyo Japan

**Keywords:** cabozantinib, cytoreductive nephrectomy, inferior vena cava tumor thrombus, metastatic papillary renal cell carcinoma, nivolumab

## Abstract

**Introduction:**

The effectiveness of nivolumab plus cabozantinib for metastatic papillary renal cell carcinoma with inferior vena cava tumor thrombus remains unclear.

**Case presentation:**

A 77‐year‐old male was diagnosed with right papillary renal cell carcinoma with a metastatic lesion on Gerota's fascia, lymph node metastasis, and inferior vena cava tumor thrombus. He was treated with nivolumab plus cabozantinib. As all lesions regressed enough to permit complete resection, radical nephrectomy, thrombectomy, and retroperitoneal lymph node dissection were performed. No viable malignant cells were identified histopathologically. Despite the discontinuation of nivolumab plus cabozantinib, there has been no recurrence for 9 months.

**Conclusion:**

Nivolumab plus cabozantinib has effectiveness for metastatic papillary renal cell carcinoma with inferior vena cava tumor thrombus.

**Abbreviations & Acronyms**
CN: cytoreductive nephrectomy
CR: complete response
CT: computed tomography
eGFR: estimated glomerular filtration rate
ICI: immune checkpoint inhibitor
IVC: inferior vena cava
IVCTT: inferior vena cava tumor thrombus
Len‐Pem: lenvatinib plus pembrolizumab
MET: mesenchymal‐epithelial transition factor
mPRCC: metastatic papillary renal cell carcinoma
mRCC: metastatic renal cell carcinoma
Nivo‐Cabo: nivolumab plus cabozantinib
PS: performance status
TKI: tyrosine kinase inhibitors

**Keynote message**

Nivolumab plus cabozantinib has effectiveness for metastatic papillary renal cell carcinoma with inferior vena cava tumor thrombus.

## Introduction

Nivo‐Cabo became one of the standard treatments for clear‐cell mRCC following the checkmate 9ER phase 3 trial^1^ and has been reported to also be effective for mPRCC.^2^, ^3^ However, the effectiveness of Nivo‐Cabo for mPRCC with IVCTT remains unclear. We encountered a patient who achieved CR of mPRCC with IVCTT to Nivo‐Cabo. This patient's clinical course can help urologists develop treatment strategies for mPRCC with IVCTT.

## Case presentation

**Fig. 1:**
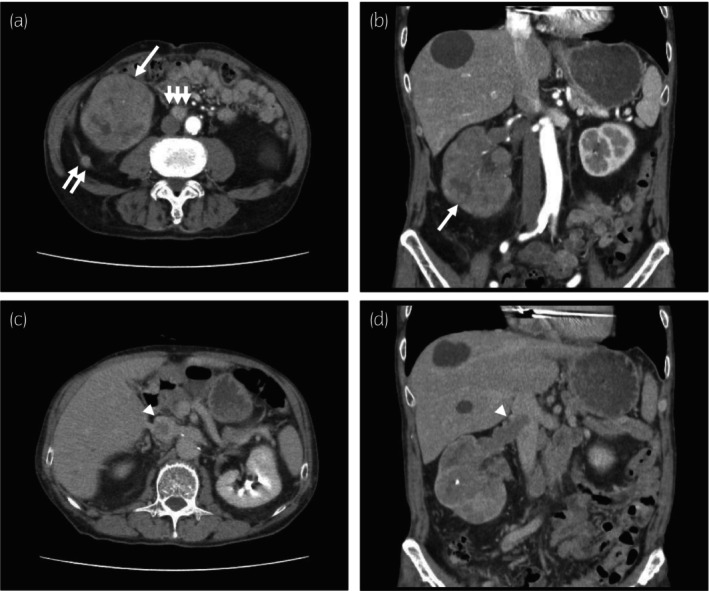
Contrast‐enhanced CT images of the renal tumor, a tumor on Gerota's fascia, lymphadenopathy, and IVCTT. (a, b) Arterial phase images of contrast‐enhanced CT. A renal mass (single arrow), mass on Gerota's fascia (double arrows), and interaortocaval lymphadenopathy (triple arrows) are shown. (c, d) IVCTT images of contrast‐enhanced CT. The tumor thrombus (arrowhead) extends from the renal vein to the IVC beneath the junction of the hepatic vein. (d) shows the cranial end of the tumor thrombus.

**Fig. 2:**
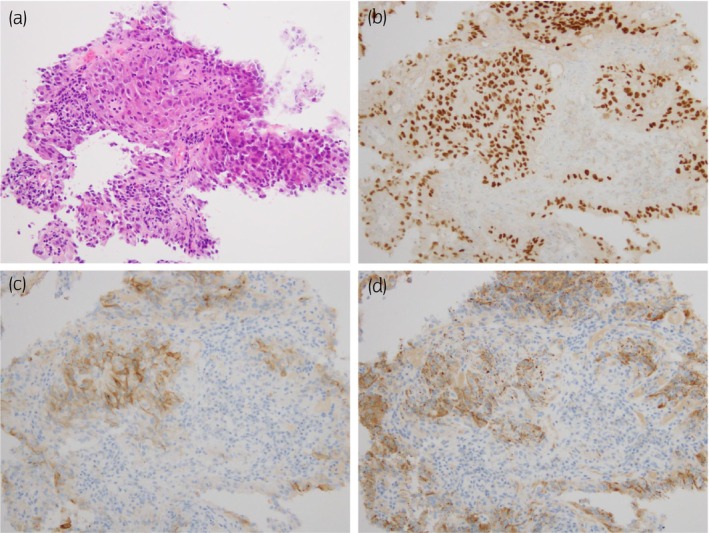
Histopathological examination findings of percutaneous needle biopsy specimens for a right renal mass. (a) An image of hematoxylin and eosin staining in 200× field of view. (b–d) Images of immunohistochemical stainings in 200× field of view. (b) PAX8, (c) CD10, and (d) AMCAR. Hematoxylin and eosin staining shows stratified malignant cells with papillary growth, which was immunoreactive for PAX8, AMCAR, and CD10. These findings are suggestive of papillary renal cell carcinoma, the histological subgroups of which were evaluated as type 2.

A 77‐year‐old male with a medical history of asthma and pacemaker insertion due to Brugada syndrome presented to our hospital with renal dysfunction. Blood tests showed renal dysfunction (creatinine: 1.15 mg/dL, eGFR: 47.9 mg/dL) and high levels of inflammation (C‐reactive protein: 19.08 mg/dL), and urine tests showed proteinuria. As screening CT revealed a mass in the right kidney, contrast‐enhanced CT was additionally performed. A 71 mm mass in the lower portion of the right kidney, a mass on Gerota's fascia, interaortocaval lymphadenopathy, and IVCTT (Neves classification level II) were revealed^4^ (Fig. 1). Percutaneous needle biopsy for the renal mass was highly suspicious for PRCC type 2 (Fig. 2), which led to a diagnosis of PRCC with Gerota's fascia metastatic lesion and IVCTT.

**Fig. 3:**
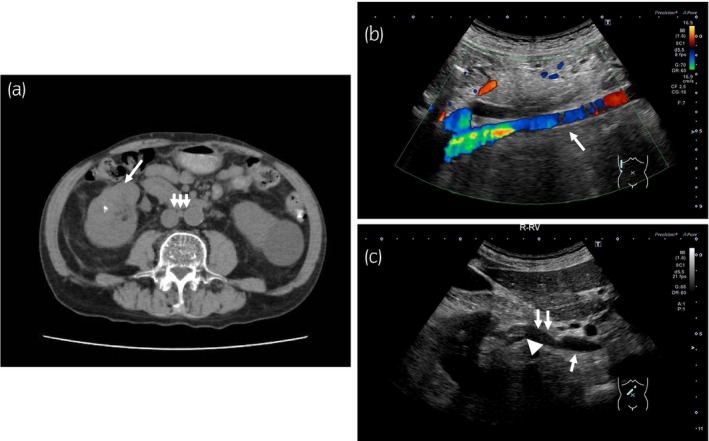
Plain CT image and ultrasonography images after 12 cycles of Nivo‐Cabo. (a) A plain CT image. (b, c) Ultrasonography images. (a) All lesions had regressed 24 weeks after the start day of Nivo‐Cabo. Though the renal mass (single arrow) and the interaortocaval lymphadenopathy (triple arrows) remain, the mass on Gerota's fascia and the tumor thrombus had regressed sufficiently so as not to show on plain CT (b, c) The tumor thrombus did not exist in the IVC (single arrow) given the normal blood flow signal. However, the tumor thrombus (arrowhead) remained within the renal vein (double arrow).

The patient's Eastern Cooperative Oncology Group PS was Grade 1, and his International Metastatic RCC Database Consortium risk was poor, given his anemia, hypercalcemia, thrombocytosis, and time from diagnosis to systemic therapy. Nivo‐Cabo was selected for primary treatment, considering the demonstration of PRCC. 4 weeks after treatment was commenced, his renal function was impaired (creatinine: 2.68 mg/dL, eGFR: 19.0 mg/dL). As the cause was suspected to be cabozantinib, the drug was suspended until the patient's renal function improved, which it failed to do. Recognizing that his renal function might be further impaired with the restart of cabozantinib, we decided to restart with an 80% dose to prolong overall survival 12 weeks after the start date. Plain CT 24 weeks after the start date showed that all lesions had regressed and IVCTT was not visible, thereby exhibiting a partial response as per the new Response Evaluation Criteria in Solid Tumors. However, additional ultrasonography showed IVCTT within the renal vein (Fig. 3).

**Fig. 4:**
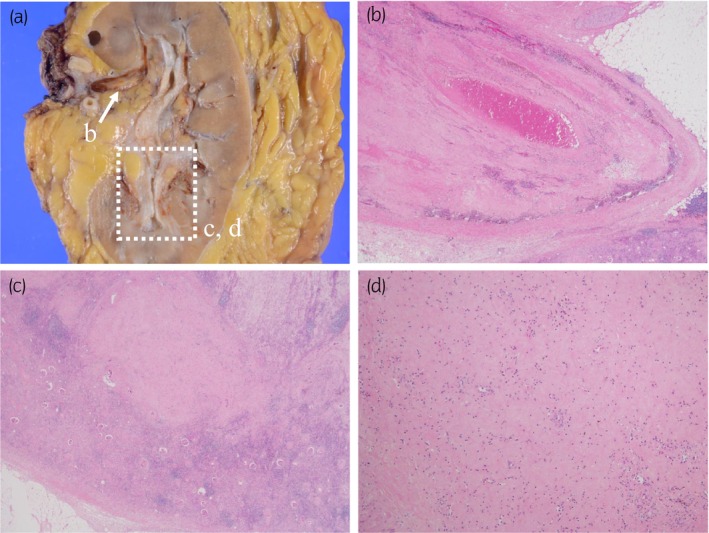
Histopathological examination findings of the resected specimens. Macroscopic images and microscopic images of hematoxylin and eosin staining of the resected specimen are presented in panels (a) and (b–d), respectively. (a) The resected specimen grossly shows that though the renal mass disappeared, the denatured tumor thrombus remained within the renal vein. (b–d) Microscopic images of the resected specimens. (b) Microscopic images of the tumor thrombus within the renal vein (white arrow in panel a) in 20× field of view. (c, d) Microscopic images of the area where the tumor would have been (enclosed by the dotted line in panel a). Images of 20× fields and 100× fields of view are presented in panel (c) and (d), respectively. No viable malignant cells were identified microscopically in the tumor thrombus and other resected specimens, and there was only scar tissue where the renal mass would have been.

Given that his PS was good and all lesions had regressed enough to resect more completely and safely, radical nephrectomy, tumor thrombectomy, and retroperitoneal lymph node dissection were performed after a 3‐week drug holiday. The duration of pre‐operative Nivo‐Cabo treatment was 6 months. All lesions were resected completely but were accompanied by adhesions, especially IVCTT. As intraoperative ultrasonography to determine the resection margin showed tumor thrombus within the renal vein, it was resected along with the IVC wall by the side‐clamp technique. However, the IVC wall was harder than normal, possibly indicating viable malignant cell infiltration to the IVC wall. Therefore, we additionally resected the IVC wall and performed intraoperative rapid pathological examination, which showed a negative margin. Reconstruction of the IVC with a patch was not performed as over 70% diameter was left. The resected specimens grossly showed that although the renal mass had disappeared, the denatured tumor thrombus remained within the renal vein. However, no viable malignant cells were identified histopathologically in the specimens (Fig. 4), suggesting the CR of mPRCC with IVCTT to Nivo‐Cabo. Although Nivo‐Cabo was discontinued postoperatively, there has been no recurrence for 9 months.

## Discussion

PRCC, which is stratified into histological (type 1 or 2) or molecular (MET‐driven or MET‐independent) subgroups, is the most common non‐clear cell renal carcinoma.^5^ Although the standard treatment for mPRCC has not been defined as no phase III trials have been reported, European Association of Urology guidelines recommend cabozantinib, pembrolizumab, Len‐Pem, and Nivo‐Cabo.^2^

While prospective studies with cabozantinib, pembrolizumab, Len‐Pem, and Nivo‐Cabo for non‐clear cell mRCC have shown efficacy to mPRCC, it remains unclear if they are effective for mPRCC with IVCTT.^3^, ^6^, ^7^, ^8^ Therefore, although the regimen selection in the present case was difficult, Nivo‐Cabo was selected as Len‐Pem was not approved in Japan at the time and Nivo‐Cabo has the highest objective response rate among them (with the exception of Len‐Pem). Consequently, the present case achieved pathological CR, suggesting that Nivo‐Cabo is effective for mPRCC with IVCTT.

Although Nivo‐Cabo was effective in our case, renal dysfunction was also observed. We suspected cabozantinib as the cause since renal dysfunction induced by ICI is rare, and the patient's renal dysfunction did not worsen despite continuing nivolumab.^9^, ^10^ However, interstitial nephritis was recognized histopathologically in the resected specimens (Figure S1), suggesting the possibility of interstitial nephritis induced by nivolumab as a cause of his renal dysfunction.

The present case also involved deferred CN for mRCC treatment. The CARMENA and SURTIME studies, which prospectively discussed CN for mRCC treated with TKI, suggested that the indication and timing of CN should be considered carefully.^11^, ^12^ There has been a paradigm shift in systemic therapy for mRCC since the advent of ICI. Although the role of CN in mRCC treated with ICIs or ICI‐TKI remains controversial,^13^, ^14^ the role of upfront CN and deferred CN for mRCC treated with ICIs or ICI‐TKI, respectively, has recently been suggested.^15^, ^16^ In our case, all lesions regressed but did not disappear on imaging modalities despite obtaining pathological CR. This finding suggests that a difference between radiological and pathological response can exist after immunotherapy, as recognized in a previous case report.^17^ We also observed this difference in a CR case of mRCC with IVCTT treated with nivolumab plus ipilimumab.^18^ Which timing of CN, immediate or deferred, better contributes to prolonging overall survival remains unknown. However, deferred CN may enable histological response evaluation and consequently aid in the formation of therapeutic strategies such as the continuation or discontinuation of systemic therapy.

Finally, the present case suggests that surgeons should pay attention to adhesions in deferred CN, especially in IVCTT cases. Presurgical TKI, ICI, and ICI‐TKI have been reported to cause intraoperative adhesions.^16^, ^19^ In the present case, the adhesion of IVCTT, which may have been caused by Nivo‐Cabo, created confusion regarding the IVC resection line. Therefore, in deferred CN cases with IVCTT, surgeons should prepare for intraoperative rapid histopathological examination in the event of confusion regarding the IVC resection line and should preoperatively consult with vascular surgeons as additional resection could require IVC reconstruction.

## Conclusion

The present case is the first to obtain CR of mPRCC with IVCTT to Nivo‐Cabo. Nivo‐Cabo has effectiveness for mPRCC with IVCTT. Deferred CN may allow better consideration of therapeutic strategies. Surgeons should pay attention to adhesions in deferred CN, especially in IVCTT cases.

## Author contributions

Michikata Hayashida: Conceptualization; writing – original draft; writing – review and editing. Yuji Miura: Supervision. Takeshi Yamaguchi: Conceptualization. Makoto Tanaka: Writing – review and editing. Taro Yamanaka: Conceptualization. Kohji Takemura: Writing – review and editing. Suguru Oka: Conceptualization. Kazushige Sakaguchi: Writing – review and editing. Shinji Ito: Writing – review and editing. Shinji Urakami: Supervision.

## Conflict of interest

Yuji Miura has received personal fees from Ono Pharmaceutical, Bristol Myers Squibb, MSD, Eisai, and Takeda that played no role in this case report.

## Approval of the research protocol by an Institutional Reviewer Board

Not applicable.

## Informed consent

Written informed consent was obtained.

## Registry and the Registration No. of the study/trial

Not applicable.

## Supporting information


**Figure S1.** Histopathological examination images of interstitial nephritis in resected specimens. (a, b) Microscopic images of hematoxylin and eosin staining of the resected specimens (panel a: 40× field of view, panel b: 200× field of view). Inflammation and fibrosis accompanying lymphocytes, plasma cells, and eosinophils were recognized along with medullary rays from the renal subcapsular area, and some parts of the normal renal tubule structure were destroyed. These findings indicate the presence of interstitial nephritis.
