# Supplementary figures and images for: Complete response of metastatic papillary renal cell carcinoma with inferior vena cava tumor thrombus to nivolumab plus cabozantinib
Source: IJU Case Rep. 2023 Sep 5;6(6):419–23. doi: 10.1002/iju5.12638 (PMC10622194; doi:10.1002/iju5.12638)

## Slide 1
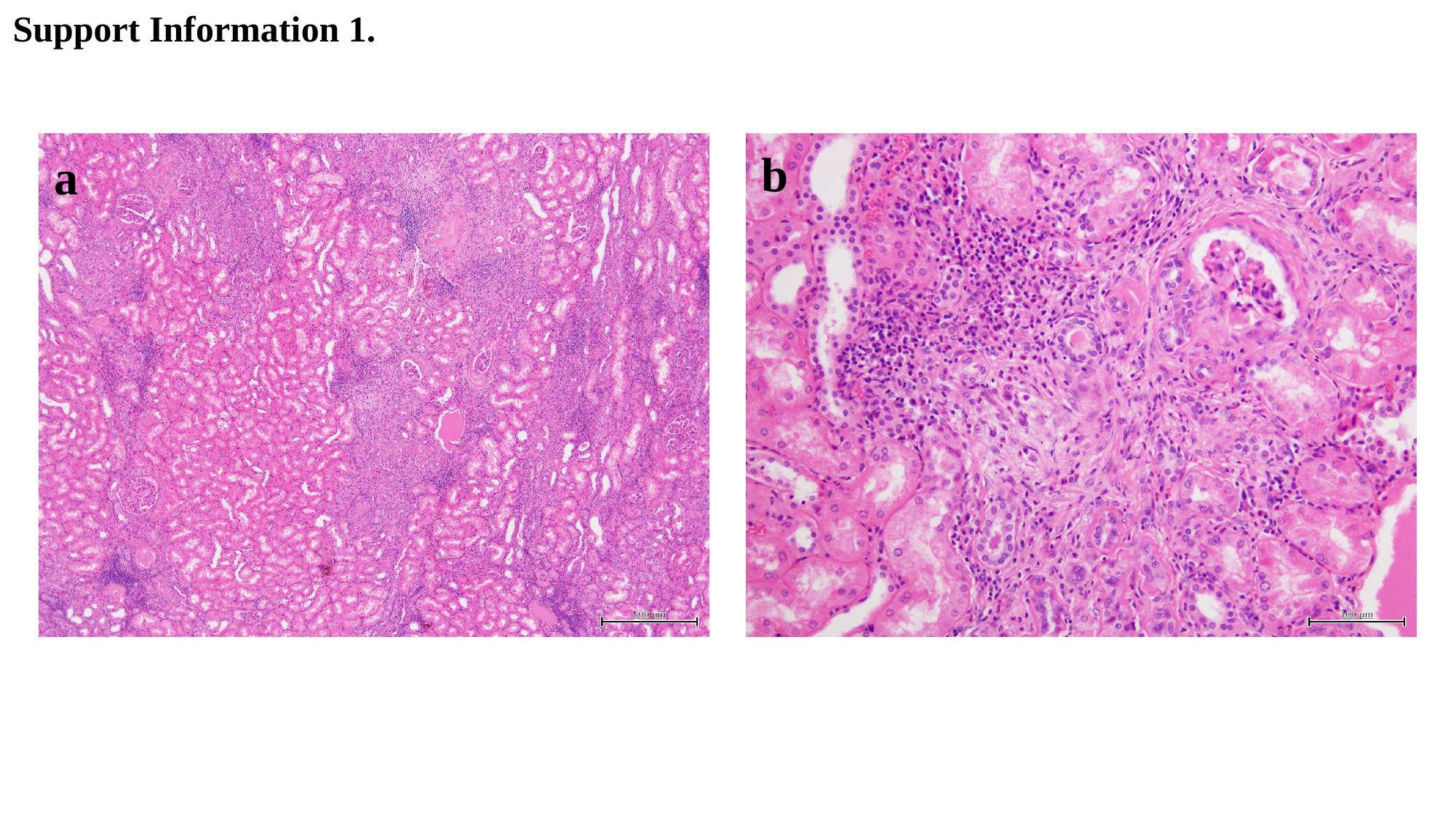

Support Information 1.
b
a

Supplement: Supplementary file 1 — Figure S1. Histopathological examination images of interstitial nephritis in resected specimens. (a, b) Microscopic images of hematoxylin and eosin staining of the resected specimens (panel a: 40× field of view, panel b: 200× field of view). Inflammation and fibrosis accompanying lymphocytes, plasma cells, and eosinophils were recognized along with medullary rays from the renal subcapsular area, and some parts of the normal renal tubule structure were destroyed. These findings indicate the presence of interstitial nephritis. [file IJU5-6-419-s001.pptx]
